# Insulin Signaling Impairment in the Brain as a Risk Factor in Alzheimer’s Disease

**DOI:** 10.3389/fnagi.2019.00088

**Published:** 2019-04-24

**Authors:** Christian Hölscher

**Affiliations:** Research and Experimental Center, Henan University of Chinese Medicine, Zhengzhou, China

**Keywords:** growth factor, brain, GLP-1, mitochondria, apoptosis, autophagy, inflammation

## Abstract

Type 2 diabetes is a risk factor for developing Alzheimer’s disease (AD). The underlying mechanism that links up the two conditions seems to be the de-sensitization of insulin signaling. In patients with AD, insulin signaling was found to be de-sensitized in the brain, even if they did not have diabetes. Insulin is an important growth factor that regulates cell growth, energy utilization, mitochondrial function and replacement, autophagy, oxidative stress management, synaptic plasticity, and cognitive function. Insulin desensitization, therefore, can enhance the risk of developing neurological disorders in later life. Other risk factors, such as high blood pressure or brain injury, also enhance the likelihood of developing AD. All these risk factors have one thing in common – they induce a chronic inflammation response in the brain. Pro-inflammatory cytokines block growth factor signaling and enhance oxidative stress. The underlying molecular processes for this are described in the review. Treatments to re-sensitize insulin signaling in the brain are also described, such as nasal insulin tests in AD patients, or treatments with re-sensitizing hormones, such as leptin, ghrelin, glucagon-like peptide 1 (GLP-1),and glucose-dependent insulinotropic polypeptide (GIP). The first clinical trials show promising results and are a proof of concept that utilizing such treatments is valid.

## Introduction

Recently, type 2 diabetes mellitus (T2DM) has been identified as a risk factor for Alzheimer’s disease (AD). Epidemiological studies of patient data sets have found a clear correlation between T2DM and the risk of developing AD or other neurodegenerative disorders (Hoyer, 1998; Luchsinger et al., 2004; Ristow, 2004; Strachan, 2005; Haan, 2006). In one study, 85% of AD patients had diabetes or showed increased fasting glucose levels, compared to 42% in age-matched controls (Janson et al., 2004). In longitudinal studies of cohorts of people, it was found that glucose intolerance was a good predictor for the development of dementia later in life (Schrijvers et al., 2010; Ohara et al., 2011; Li T.C. et al., 2017). In the Hisayama study, a total of 1,017 dementia-free subjects aged ≥60 years were tested for glucose tolerance and followed up for 15 years. It was found that the glucose intolerance correlated well with the development of vascular dementia and AD in later life (Ohara et al., 2011).

When analyzing the brain tissue of AD patients, it was observed that insulin signaling was much desensitized, even in AD patients that did not have T2DM (Frolich et al., 1998). One study found that the levels of insulin, IGF-1 and IGF-II were much reduced in brain tissue. In addition, levels of the insulin receptor, the insulin-receptor associated PI3-kinase, and activated Akt/PKB kinase were much reduced (Steen et al., 2005). A second study found increased levels of IGF-1 receptors and the localization of insulin receptors within cells rather than on the cell surface where they could function. Decreased levels of insulin receptor substrates IRS-1 and IRS-2 levels were observed in neurons, and increased levels of inactivated phospho (Ser^312^)IRS-1 and phospho (Ser^616^)IRS-1 (Moloney et al., 2010). A third study analyzed the biochemical changes in the brains of AD patients in great detail, and also analyzed the functionality of insulin signaling using an *ex vivo* insulin incubation technique. In this study, tissue from the hippocampal formation was incubated with insulin to measure any biochemical changes. It was found that the second messenger cascade activated by insulin was significantly impaired. For example, the activation of the downstream kinase Akt/PKB at the Serine^473^ site was almost completely abolished, while the activation was effective in age-matched control brain tissue (Talbot et al., 2012).

## Insulin Is a Key Growth Factor

**FIGURE 1:**
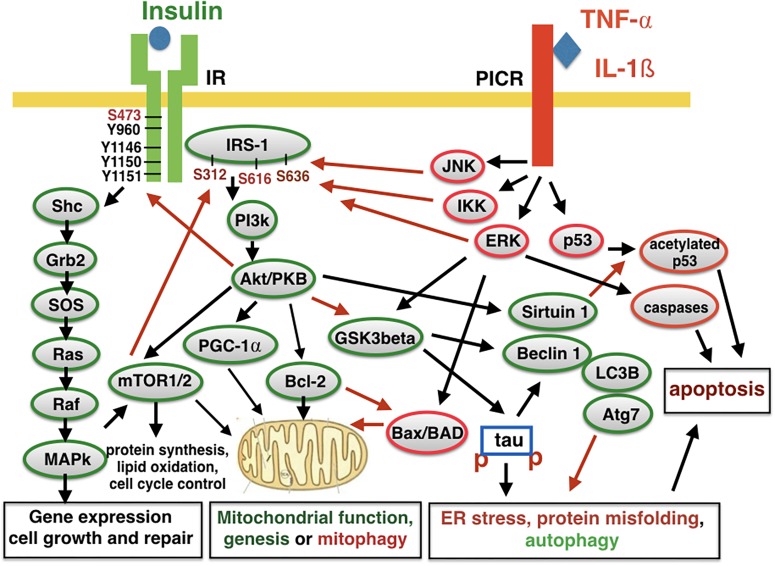
Insulin signaling and pro-inflammatory signaling counteract each other. The activation of the insulin receptor (IR) leads to auto-phosphorylation and activation. Several other kinases can activate or inactivate the receptor. The insulin-receptor substrate 1 (IRS-1) also contains several phosphorylation sites that can activate or inhibit second messenger signaling. Downstream signaling activates key physiological processes such as energy utilization, mitochondrial function and replacement, protein synthesis, autophagy, and inhibiting autophagy. Activating pro-inflammatory cytokine receptors (PICR) counteracts these processes and enhances mitophagy, autophagy, and apoptosis. Kinases such as JNK and IKK can phosphorylate IRS-1 to inhibit insulin signaling and induce insulin de-sensitization. IL-1ß, interleukin 1ß; TNF-α, tumor necrosis factor a; JNK, NH2-terminal c-Jun kinase; IKK, inhibitor of kappa B kinase; ERK, extracellular regulated kinase; MAPK, mitogen activated protein kinase; SHP-2, Src-like homology 2(SH2) domain containing protein tyrosine phosphatase; PGC-1α, peroxisome proliferator-activated receptor γ coactivator 1-α; Grb2, growth factor receptor binding protein 2; SOS, son of sevenless protein; PI3K, phosphatidylinositol 3-kinase; IRS, insulin receptor substrate; PKB, protein kinase B,also known as Akt; Raf, regulation of alpha-fetoprotein; Ras, rat sarcoma virus peptide; LC3B, microtubule-associated proteins 1A/1B light chain 3B; Atg7, autophagy-related protein 7; Bcl-2, B-cell lymphoma 2; Bad, Bcl-2-associated death promoter; Bax, Bcl-2 associated X protein; Shc, Src homology collagen peptide. Red arrows, inhibiting activity; Black arrows, activating activity.

Insulin is not just a hormone that regulates blood glucose levels, but it is also an important growth factor that regulates neuronal growth, repair, and functions. The insulin receptor (IR) activates gene expression via MAPkinase and Akt/PKB cell signaling that enhances glucose uptake, mitochondrial function and replacement, protein synthesis, autophagy, and the inhibition of apoptosis (Carro and Torres-Aleman, 2004; Schubert et al., 2004; Heras-Sandoval et al., 2014; Holscher, 2014; Najem et al., 2014; see Figure 1). Among other key physiological functions, it also activates the Nrf2 promotor to activate gene expression that protects cells against oxidative stress (Song et al., 2018). Synaptic activity in the brain and the integrity of neuronal networks is also regulated by insulin (Ferrario and Reagan, 2018). It is, therefore, plain to see how the reduction of insulin signaling in the brain impairs cell maintenance and repair and puts neurons at risk to develop neurodegenerative disorders over time. As neurons in the brain are not replaced, the damage will accumulate and may present itself as AD in old age.

## Causes for Insulin Desensitization

As there are few genetic links to AD, the disease is predominantly a “sporadic”condition that is triggered by environmental influences. Only around 1–5% of cases can be linked to a clear genetic origin, such as a mutation in the amyloid precursor protein (APP) or presenilin-1 gene (Blennow et al., 2006; Guerreiro and Hardy, 2014; Karch and Goate, 2015). The key question is: what else causes AD and what are the drivers of disease progression?

### Chronic Inflammation

Under physiological conditions, there is no inflammation response in the brain. However, chronic exposure to inflammatory triggers can induce a chronic inflammation response in the brain that is detrimental for neuronal health and survival. Chronic neurodegenerative disorders are always accompanied by a robust chronic inflammation response, and there is an agreement that this response is a key driver for neurodegenerative disorders (Akiyama et al., 2000; Perry et al., 2007; Lee et al., 2010; Clark and Vissel, 2018). The inflammation response activates microglia in the brain and enhances the release of oxidative stressors, such as nitric oxide and pro-inflammatory cytokines which inhibit growth factor signaling (Cunningham et al., 2005; Vaz et al., 2011). It is of interest to note that key risk genes for AD are linked to the control and progression of the inflammation response. The APOEe4 allele is a major risk factor for developing AD (Blacker et al., 1997). APOE is a pro-inflammatory cytokine that is released by activated microglia (Krasemann et al., 2017). APOE can inhibit receptors of the reelin family that activate intracellular signaling similar to the IR and that modulate glucose uptake, energy utilization, cell growth, and synaptic plasticity, as well as reduce the inflammation response (Herz and Chen, 2006; Lane-Donovan et al., 2014; Liu et al., 2015). The APOEe4 isoform appears to be more effective in blocking these receptors, leading to a loss of growth factor signaling and reduced cell growth, repair and metabolism, and additionally reduced inhibition of inflammation (Krasemann et al., 2017). It is easy to see how such actions could enhance the risk of developing AD, in particular if the growth factor insulin has lost its effectiveness. APOEe4 was even found to inhibit IR activity and enhance the uptake of the receptor in the cell (Zhao et al., 2017). When analyzing plasma samples of AD patients, it was shown that in APOEe4 allele carriers, microglia activation markers were different from those that carry the APOEe2 allele, and in non-demented controls. The marker CD68 was found to be increased in APOEe4 allele carriers, and this correlated with a poorer outcome in the Mini-Mental State Examination compared to carriers of the APOEe2 allele. APOEe2 allele carriers showed enhanced levels of Iba-1 microglia markers which correlated with a better performance in the in the Mini-Mental State Examination (Minett et al., 2016). Furthermore, APOEe4 carriers showed enhanced plasma levels of TNF-α and reduced Akt/PKB activity (Morris et al., 2018). These observations support the concept that AD progression is linked to reduced growth factor activity and a reduced inhibition of the inflammation response.

Another important risk factor for AD is the gene for TREM2, an immune phagocytic receptor expressed on brain microglia that regulates phagocytosis and the inflammation response. Homozygous mutations in TREM2 cause Nasu-Hakola disease, a rare recessive form of dementia (Jin et al., 2014). TREM2 is a main regulator of the activation of microglia in the inflammation response (Konishi and Kiyama, 2018). In carriers of TREM2 mutations, the inflammation response is much enhanced, and pro-inflammatory cytokines are increased (Roussos et al., 2015). Importantly, APOE and TREM2 interact in the regulation of the inflammation response, further emphasizing the key role of inflammation in AD progression (Morris et al., 2018; Shi and Holtzman, 2018).

Insulin reduces the chronic inflammation response by inhibiting secondary cell signaling induced by pro-inflammatory cytokines (Bamji-Mirza et al., 2014; Iloun et al., 2018; Figure 1). A de-sensitization of insulin signaling enhances the inflammation response and the de-sensitization observed in T2DM (Biessels et al., 2002), therefore, not only compromises growth factor signaling, and energy utilization in the brain, but also facilitates the chronic inflammation response.

### Triggers for Chronic Inflammation in the Brain

The chronic inflammation response observed in neurodegenerative disorders is triggered initially and maintained by continuous stress to the system. Ordinarily, the inflammation response would be terminated after the initial event that triggered it had stopped. Due to the nature of neurodegenerative disorders, such as AD, the stressor persists and the inflammation response becomes chronic. Mutations in the APP gene are known to cause early-onset familial forms of AD (Hardy, 1997; Hutton et al., 1998; Blennow et al., 2006). The aggregated or misfolded amyloid protein that results from this can induce an inflammation response (Lourenco et al., 2013), most likely by activating Toll-like receptors (Clark and Vissel, 2018). In animal models that express human mutated APP, a chronic inflammation response is observed in the brain (McClean and Holscher, 2014a; Olmos-Alonso et al., 2016). The chronic exposure to such low-level inflammation stress could lead to AD. It is important to note in this context that the mutated APP gene does not cause AD but accelerates its development. This could explain why the removal of amyloid plaques from the brains of AD patients did not stop disease progression (Holmes et al., 2008). By the time the amyloid is removed, the chronic inflammation response has already been firmly established and continues to expose neurons to oxidative stress and pro-inflammatory cytokines. Auto-immune conditions are also under discussion as drivers for AD. It is straight-forward to see that an auto-immune response in the brain could start chronic inflammation responses which eventually lead to AD (Korczyn and Vakhapova, 2007; Lehrer and Rheinstein, 2015). The NIH decided to fund further investigations of this link with AD (McCarthy, 2014).

Viral infection is another possible way of inducing a chronic inflammation response in the brain. Several studies show that viral infection can cause neurodegeneration and encephalitis if it infiltrates the brain (Damasio et al., 1989; Hogestyn et al., 2018). A recent study demonstrated that the Herpes virus was present in brain tissue of AD patients (Readhead et al., 2018). It is, however, not clear whether the virus was the cause of AD or if the infection occurred when the disease was already established, and the immune system had been compromised. It is, however, feasible that in some cases, the infection of the brain in vulnerable people induces a chronic inflammation response that can eventually lead to AD (Itzhaki et al., 2004a). The same model applies to infection with bacteria such as *Chlamydia pneumoniae*, which has been identified in brain tissue of people with AD (Itzhaki et al., 2004b).

High blood pressure is another risk factor for developing AD (Marfany et al., 2018). Again, enhanced inflammation in the brain has been found in people with high blood pressure (Wenzel, 2018). Stroke and hemorrhage in the brain is another risk factor for AD. The induction of an inflammation response in the brain has been well established (de Oliveira Manoel and Macdonald, 2018).

T2DM not only desensitizes insulin signaling in the brain (Blazquez et al., 2014), it also enhances inflammation markers such as levels of pro-inflammatory cytokines (Herder et al., 2013; Illien-Junger et al., 2013; Denver et al., 2018). Importantly, activated microglia are also found in the brains of people with T2DM, paving the way for chronic inflammation in the CNS that could lead to AD (Maldonado-Ruiz et al., 2017).

This short list of potential risk factors for AD that trigger an inflammation response is by no means complete. It demonstrates that there may be many initial triggers that could result in the development of AD in later life, depending on additional factors such as the individual vulnerability to these triggers and the inability of the brain to bring the inflammation response under control. For example, possession of the APOEe4 allele increases the vulnerability to viral infections in the brain (Itzhaki et al., 2004b).

## Re-Sensitizing Insulin Signaling in the Brain to Prevent Ad

### Treating AD Patients With Insulin

As insulin signaling has been identified as a feature of AD pathology, several strategies have been tested to find out if re-sensitizing insulin signaling in the brains of AD patients may be promising. Treating patients with insulin via nasal application to increase transfer into the brain, while reducing peripheral effects of insulin, has shown very promising effects in several clinical trials (Craft et al., 2013; Freiherr et al., 2013).

A pilot study testing nasal application of insulin in MCI/AD patients tested 26 memory-impaired subjects and 35 controls. The study had a double blind and randomized patient allocation design and a placebo group. Importantly, the insulin treatment had no effect on plasma insulin or glucose levels. Insulin treatment showed a better effect in memory-impaired non-APOEe4 carriers than for memory-impaired APOEe4 carriers and control subjects (Reger et al., 2006). A second double blind pilot study tested 24 AD/MCI patients. The insulin-treated group showed improved memory for verbal information after a delay compared to the placebo group, and furthermore improved the amyloid 40/42 ratio (Reger et al., 2008b).

In a third study, 33 AD/MCI patients and 59 control subjects received five intranasal treatments of insulin or placebo which improved recall of verbal memory in memory-impaired non-APOEe4 carriers. However, memory-impaired APOEe4 carriers displayed a decline in verbal memory. Drug treatment also improved plasma amyloid-beta levels in memory-impaired subjects and controls depending on APOE genotype status (Reger et al., 2008a). These results demonstrate a complex interaction between insulin treatment and APOEe allele status. As APOE plays an important role in inflammation, and chronic inflammation is an important aspect of AD disease progression, it is easy to see that different alleles can have facilitating or inhibiting effects on AD development. APOEe4 appears to enhance the inflammation response (McGeer et al., 1997). It acts in conjunction with TREM2 to regulate microglia activation in the brain (Morris et al., 2018; Shi and Holtzman, 2018). This could explain why carriers of the APOEe4 allele have an increased risk of developing AD. As insulin can also activate an inflammation response at higher concentrations, most likely as a negative feedback mechanism to avoid excessive energy utilization (Tsatsoulis et al., 2013; Maldonado-Ruiz et al., 2017), the dosing of insulin and the state of insulin de-sensitization in the AD patients will be crucial to determine the outcome of insulin treatment.

A larger randomized, double-blind, placebo-controlled clinical pilot trial tested 104 patients with either MCI or mild to moderate AD. Patients received either placebo or one of two doses of insulin for 4 months. Primary measures consisted of delayed story recall score and the Dementia Severity Rating Scale score, and secondary measures included the Alzheimer Disease’s Assessment Scale-cognitive subscale (ADAS-cog) score and the Alzheimer’s Disease Cooperative Study-activities of daily living (ADCS-ADL) scale. ^18^FDG-PET brain scans were conducted before and after treatment and showed a clear protection of cortical activity in AD patients. This scan measures glucose uptake and activity of neurons in the brain. Both insulin doses preserved general cognition and functional abilities assessed by care givers. Episodic memory was improved and changes were still present 2 months after cessation of treatment (Craft et al., 2012).

Another trial in AD/MCI patients tested the insulin analog detemir (Levemir) for 3 weeks. Treated patients improved in memory performance compared to a placebo group, but only if they had high levels of insulin resistance at baseline. Detemir is more effective than insulin and remains in the body for up to 24 h. Surprisingly, neither dose of insulin was more effective than placebo in memory tests. However, when patients in each treatment arm were grouped into those with low versus high insulin resistance, both groups differed from placebo controls. Patients with high insulin resistance showed an improvement in the memory tests when treated with detemir, compared to an increase of less than 0.1 point in the placebo patients with high insulin resistance. The patients with low insulin resistance treated with detemir had a bigger decrease in memory tests than the placebo group. Carriers of the APOEe4 allele were benefiting from detemir treatment, while non-carriers showed worsened memory compared to placebo (Claxton et al., 2013, 2015; Craft et al., 2013). A double blind, placebo-controlled study tested the effects of 4 months of nasal insulin or detemir treatment. Interestingly, the detemir group did not show improvements, while the insulin-treated group showed preserved memory performances and brain tissue volumes in MRI scans. An improved ration of phosphorylated tau to amyloid 42 was also observed in CSF samples (Craft et al., 2017).

These different clinical trials demonstrate that overcoming insulin resistance in the brain has protective effects in MCI/AD patients and shows improvements in cognition and relevant biomarkers. It is a proof of concept that insulin desensitization is instrumental in driving cognitive impairments in AD, and that an improvement of insulin signaling has genuine benefits.

### Strategies to Re-sensitize Insulin Signaling in the Brain

#### Metformin

Several treatment strategies exist to improve insulin signaling in people with T2DM. A standard drug prescribed to patients is metformin, which can re-sensitize insulin signaling in diabetes. However, when comparing metformin with GLP-1 receptor agonists in animal models of neurodegeneration, metformin is far less protective (Gault and Holscher, 2018). In a clinical trial testing metformin in patients with AD, the drug showed no protective effect. Treatment for 12 months only showed a very small improvement in a recall test with no changes in ADAS-cog or in ^18^FDG-PET brain scans (Luchsinger et al., 2016). While metformin is widely used in the clinic, it is actually not a very potent drug and more effective drugs will have to be given as T2DM progresses (Lentferink et al., 2018). Epidemiological studies report conflicting results with some showing a reduction of the risk to develop AD when taken over years (Hsu et al., 2011; Ng et al., 2014), while other studies showed an increase in the risk of developing AD after long-term use (Imfeld et al., 2012; Kuan et al., 2017). In a direct comparison with other drugs that enhance insulin sensitivity, metformin was inferior to more potent drugs such as GLP-1 analogs (Lennox et al., 2014). The reason for this may be that metformin only activates the AMP kinase, which is activated during starvation, but not the growth-factor associated PI3kinase (Turban et al., 2012). AMP kinase activates key survival genes while blocking energy expenditure on routine housekeeping activity in the cell (Hardie et al., 2012). PI3kinase, in contrast, is activated by growth factors such as insulin and IGF-1 and activates all housekeeping genes, ensuring cell growth, and repair (Talbot et al., 2012; Craft et al., 2013; Holscher, 2014). Therefore, it is easy to see that metformin is only helpful for the short term.

#### Leptin

Leptin is a hormone and growth factor that regulates energy utilization in cells, similarly to insulin. Both leptin and the leptin receptor are expressed in the brain where they play a range of roles. Importantly, some leptin receptor isoforms are linked to second messenger signaling cascades that also activate IRS, PI3k, and Akt/PKB, as insulin receptors do (see Figure 1), and therefore, can control energy utilization and cell growth on a similar level to insulin (Sweeney, 2002; Marwarha and Ghribi, 2012; Paz-Filho et al., 2012). Leptin can re-sensitize insulin signaling by interacting with the IR and signaling pathway (Fruhbeck, 2006; German et al., 2010; Mantzoros et al., 2011; Paz-Filho et al., 2012). Leptin can enhance memory formation and upregulate synaptic plasticity in the hippocampus (Harvey et al., 2006). In animal models of AD, leptin showed protective effects, and improved performances in learning tasks (Greco et al., 2010; Perez-Gonzalez et al., 2011; Sharma and Hölscher, 2014).

As leptin is linked to glucose uptake and energy utilization in cells, it is not surprising that leptin signaling desensitizes in T2DM, just as insulin signaling does (Kautzky-Willer et al., 2001; Carey et al., 2003; Cummings, 2013; Sharma and Hölscher, 2014). This observation convinced researchers studying T2DM not to develop drugs that act on the leptin receptor as potential treatments for diabetes (Mantzoros et al., 2011; Chou and Perry, 2013). There is also evidence that leptin signaling is de-sensitized in AD patients, most likely driven by the chronic inflammatory response in the brain (Lieb et al., 2009; Clark et al., 2011; Bonda et al., 2014; Khemka et al., 2014). This would make it difficult to use leptin to re-sensitize insulin signaling in the brains of AD. One proposal to circumvent the problem is to co-apply different hormone analogs that can re-sensitize leptin signaling (Sadry and Drucker, 2013).

The story does not end here, though. Leptin also plays a role in pro-inflammatory signaling. The receptor is expressed on cell types that play key roles in the inflammation response (Procaccini et al., 2012). As a hormone and growth factor, it stimulates the activity of macrophages, neutrophils, and natural killer cells (Matarese et al., 2005). It also activates the proliferation of T helper cells and the differentiation of CD4+ T-cells into inflammatory Th-17 cells (Lord et al., 1998). The transgenic models of obesity that have mutations in leptin and the leptin receptor genes, ob/ob and db/db, show enhanced inflammatory responses (Lord et al., 2001). In the animal model of multiple sclerosis (MS), an auto-immune response is induced. When trying to induce the auto-immune response in ob/ob mice, the response is much reduced, and low levels of pro-inflammatory cytokines are expressed (Matarese et al., 2001b). When replacing the missing leptin in the ob/ob mice, a full-blown auto-immune reaction is induced, and levels of pro-inflammatory cytokines are much increased (Matarese et al., 2001a). Removing leptin in wild type mice also makes them less susceptible to developing the MS auto-immune disease phenotype (Ouyang et al., 2014). As chronic inflammation plays a key role in AD, it is therefore of benefit for leptin signaling to be de-sensitized. Therefore, leptin treatment may not be beneficial as a treatment for neurodegenerative disorders (de Candia and Matarese, 2018).

#### Glucagon-Like Peptide 1 (GLP-1) and Glucose-Dependent Insulinotropic Polypeptide (GIP)

Glucagon-like peptide 1 and Glucose-dependent insulinotropic polypeptide are incretin hormones that signal high energy status in the body, similar to insulin and leptin (Lund et al., 2011). They activate membrane standing G-protein coupled receptors that are members of the glucagon-type growth factor receptor family. Classic growth factor signaling cascades are activated by the receptors, including the cAMP-PKA-CREB pathway that enhances cell growth, cell repair and expression of insulin, and insulin receptors (Baggio and Drucker, 2007; Doyle and Egan, 2007; Hölscher, 2016). The PI3k-Akt/PKB-mTOR pathway is also activated, compensating for the loss of insulin signaling (Hölscher, 2018). AMPk is also activated by the receptors (Chang et al., 2018). GLP-1 enhances insulin release making it an attractive treatment for T2DM (Baggio and Drucker, 2007; Doyle and Egan, 2007; Long-Smith et al., 2013). GLP-1 receptor agonists have been developed that have an enhanced biological half-life in the blood stream. Several are currently on the market to treat diabetes (Campbell and Drucker, 2013; Elkinson and Keating, 2013; Lean et al., 2014). GLP-1 mimetics re-sensitize insulin signaling in the brains of animal models of AD (Bomfim et al., 2012; Long-Smith et al., 2013; Shi et al., 2017) and in patients with T2DM (Zander et al., 2002).

Glucagon-like peptide 1 mimetics showed clear neuroprotective effects in several animal models of AD (Li et al., 2010). The GLP-1 mimetic Liraglutide reduced AD hallmarks such as amyloid plaque load, memory loss, synapse loss, impaired synaptic transmission (LTP), and the chronic inflammation response in the brain (McClean et al., 2011, 2015; McClean and Holscher, 2014a). The GLP-1 mimetic lixisenatide showed similar neuroprotective effects in the APP/PS1 mouse model of AD (McClean and Holscher, 2014b). Furthermore, liraglutide reduced tangles and phosphorylated tau levels in the human P301L mutated tau gene expressing mouse, a model of fronto-temporal lobe dementia (Hansen et al., 2015). Liraglutide reduced insulin de-sensitization and reduced the inflammation response caused by the injection of amyloid oligomers into the brains of cynomologous monkeys (Lourenco et al., 2013; Batista et al., 2018). GLP-1 mimetics protect mitochondrial activity and enhance mitochondrial genesis by reducing apoptotic BAX/BAD signaling and by increasing pro-mitochondrial Bcl-2 and PGC-1a signaling (Li et al., 2016a; Shi et al., 2017; Chang et al., 2018; Wang et al., 2018). Importantly, GLP-1 mimetics protect against ER stress and normalize autophagy impairments found in neurodegenerative disorders, which can explain how amyloid or tau aggregates are removed by the activation of the GLP-1 receptor (Sharma et al., 2013; Panagaki et al., 2017).

A pilot clinical trial in AD patients demonstrated an increased glucose uptake in the brain after treatment with liraglutide (Gejl et al., 2016). Unfortunately, the trial was underpowered and did not show any results in other measures. A phase II clinical trial with 200 AD patients is currently ongoing. Patient recruitment has finished, and the results should become available late 2019 (clinical trial identifier NCT01843075). In a double-blind, placebo-controlled phase II clinical trial in PD patients, the GLP-1 receptor agonist exendin-4 showed good protective effects compared to placebo, even after 3 months of wash-out of the drug (Athauda et al., 2017). Further clinical trials testing other GLP-1 mimetics are currently ongoing.

The sister hormone GIP also has neuroprotective properties in different animal models of neurodegenerative disorders. Stable analogs such as D-ala^2^-GIP or N-glyc-GIP reversed impairments of synaptic plasticity induced by amyloid (Gault and Holscher, 2008). The GIP analog D-Ala^2^-GIP showed neuroprotective effects in the APP/PS1 mouse model of AD by protecting memory formation, synaptic plasticity, reducing the amyloid plaque load and the chronic inflammation response in the brain, reducing oxidative stress, and normalizing neurogenesis in the dentate gyrus (Duffy and Holscher, 2013; Faivre and Holscher, 2013a,b). GIP injection ip., had protective effects on spatial learning in memory tasks and also reduced plaque formation and amyloid load in a different AD mouse model (Figueiredo et al., 2010). See (Ji et al., 2016; Verma et al., 2018) for a review.

In the 1-methyl-4-phenyl-1,2,3,6-tetrahydropyridine (MPTP) mouse model of PD, GIP analogs showed good protective effects. Motor activity was partly rescued, and the number of dopaminergic neurons in the substantia nigra was increased. The cAMP/PKA/CREB growth factor second messenger pathway was shown to be activated by the drug. The increased levels of expression of alpha-synuclein in the brain induced by MPTP were reduced by the drug. In addition, drug treatment reduced chronic neuroinflammation, oxidative stress and lipid peroxidation, and increased the expression of the growth factor BDNF (Ji et al., 2016; Li et al., 2016c; Li Y. et al., 2017; Verma et al., 2017, 2018).

Novel dual GLP-1/GIP receptor agonists also show good effects in different animal models of neurodegenerative disorder (Hölscher, 2018).

#### Ghrelin

Ghrelin is a member of the hormones and growth factors that signal energy status in the body. It is released by stomach cells during times of low food availability. It is considered the main factor that drives the health-improving effects of fasting/caloric restriction (Gomez et al., 2009; Bayliss et al., 2016b). Ghrelin does not re-sensitize insulin as it is active at low plasma glucose levels, while insulin works “against” ghrelin and is only active at high glucose levels. However, ghrelin activates a range of growth-factor signaling pathways that can compensate for a loss of insulin signaling. Ghrelin has been shown to have impressive protective effects in animal models of Parkinson’s disease. Administration of ghrelin reduced the neurodegeneration observed in the MPTP model of PD. Ghrelin reduced the MPTP-induced loss of substantia nigra (SN) dopamine neurons and striatal dopamine turnover. Ghrelin activated AMPk and ACC- driven lipid-oxidation in the striatum. Mitochondrial activity and genesis were improved. Importantly, the chronic inflammation response in the brain was much reduced by the drug (Bayliss et al., 2016a,b; Morgan et al., 2018). These impressive effects make ghrelin a candidate to be a novel treatment for PD. However, there are downsides of this strategy.

One of the main modes of action is the activation of AMP-kinase (AMPk) by the Ghrelin receptor. AMPk is a kinase that is activated under conditions of low energy, such as low cellular ATP and high AMP levels. AMPk is a key regulator of a range of physiological processes that enhance cell survival. Glucose uptake and oxidation, as well as activation of fat reserves, uptake of fatty acids and lipid oxidation to generate ATP, are increased. Importantly, AMPK upregulates genes involved in oxidative metabolism and oxidative stress resistance by regulating transcription factors of the abnormal dauer formation 16 (DAF-16)/forkhead box O (FOXO) family. This enables cells to deal with enhanced oxidative stress, which will increase in conditions of low energy and failing mitochondrial activity (Halliwell, 2006; Hardie et al., 2012). In brief, catabolic pathways are much enhanced by ghrelin signaling to ensure sufficient energy supply for cells, while anabolic pathways that are energy demanding are reduced. These include protein synthesis and ribosomal RNA (rRNA) synthesis, triglyceride synthesis, cholesterol synthesis, transcription of gluconeogenic enzymes, and others (Hardie et al., 2012). It is easy to see that such a strategy will improve survival in lean times by focusing energy expenditure on the core processes for survival. However, it is not sensible to inhibit protein synthesis over long periods of time. In particular, in neurodegenerative disorders, repair mechanisms that require de novo protein synthesis are of major importance. A first clinical trial of a ghrelin analog also points to major issues that cannot be easily inferred from animal studies. The survival time of ghrelin in the blood stream is short, but long-acting protease-resistant analogs have been developed for the clinic. Relamorelin is a pentapeptide and analog of ghrelin with improved potency and pharmacokinetics. In humans, relamorelin produces increases in plasma growth hormone, prolactin, and cortisol levels, and increases appetite. A multi- centre, randomized, double-blind, placebo-controlled study of relamorelin in patients with PD had to be terminated, as most patients experiencedchronic and severe constipation, which is another side effect of ghrelin. Only 18 out of 56 subjects completed the trial, in part because of multiple partially complete bowel movements in constipated PD patients, which made many subjects ineligible for further participation (Parkinson Study Group, 2017). Such drastic side effects do not bode well, and long-term effects of drug treatment have yet to be investigated. However, the investigation of the effects of ghrelin enriched out knowledge of underlying mechanisms of neuronal pathology and neuronal protection, which may be utilized in other forms and treatment strategies.

#### DPP-IV Inhibitors

Currently, inhibitors of the enzyme protease dipeptidyl-peptidase IV (DPP-IV) are on the market treat T2DM (Ahren, 2007). DPP-IV degrades GLP-1, GIP and other peptide hormones. Inhibition of DPP-IV therefore can enhance the blood levels of these hormones and can treat T2DM effectively. Inhibitors of DPP-IV also have protective effects in animal models of AD (Li et al., 2016b). However, DPP-IV inhibitors are small molecules that do not readily cross the BBB. The DPP-IV inhibitor saxagliptin (SAX) also showed good neuroprotective effects in the ICV. STZ animal model of AD that models insulin desensitization in the brain. Here, animals were orally administered at 0.25, 0.5, or 1 mg/kg for 60 days after treatment with STZ. The highest dose of 1mg/kg showed good protection in memory tests and lowered key biomarkers for AD and cellular stress (Kosaraju et al., 2013). In a similar study from the same lab, Vildagliptin also demonstrated neuroprotective effects. However, the effective dose was more than 10 times higher with lower protective efficiency, suggesting that Vildagliptin does not cross the BBB as readily as SAX. In addition, the protective effects of DPP-IV inhibitors in animal models are limited compared to GLP-1 receptor agonists (Abdelsalam and Safar, 2015; Darsalia et al., 2016). This is most likely due to the fact that DPP-IV inhibitors work indirectly by reducing peptide hormone cleavage, while GLP-1 receptor agonists act directly on the receptors.

## Conclusion

The evidence presented here demonstrates that insulin de-sensitization contributes to the disease progression in AD, and that the use of insulin re-sensitizing agents show promise to make a difference and to reduce disease progression. First clinical trials have shown promising results and suggest that the protective effects observed in animal studies translate to the clinic. This strategy shows great promise and hopefully will produce effective novel treatments for AD and other chronic progressive neurodegenerative disorders.

## Author Contributions

CH wrote the manuscript.

## Conflict of Interest Statement

CH is a named inventor on several patent applications that cover the use of dual GLP-1 and GIP receptor agonists to treat neurodegenerative disorders.
